# Rare serovars of non-typhoidal *Salmonella enterica* isolated from humans, beef cattle and abattoir environments in Nigeria

**DOI:** 10.1371/journal.pone.0296971

**Published:** 2024-01-22

**Authors:** Mabel Kamweli Aworh, Pernille Nilsson, Beverly Egyir, Felicia Amoa Owusu, Rene S. Hendriksen

**Affiliations:** 1 Department of Population Health and Pathobiology, College of Veterinary Medicine, North Carolina State University, Raleigh, North Carolina, United States of America; 2 Nigeria Field Epidemiology and Laboratory Training Programme, Abuja, Nigeria; 3 Research Group for Global Capacity Building, National Food Institute, WHO Collaborating Centre (WHO CC) for Antimicrobial Resistance in Foodborne Pathogens and Genomics, FAO Reference Laboratory (FAO RL) for Antimicrobial Resistance, European Union Reference Laboratory for Antimicrobial Resistance (EURL-AR), Technical University of Denmark, Kongens Lyngby, Denmark; 4 Department of Bacteriology, Noguchi Memorial Institute for Medical Research, College of Health Sciences, University of Ghana, Accra, Ghana; Universidad San Francisco de Quito, ECUADOR

## Abstract

**Introduction:**

*Salmonella* is considered one of the most significant pathogens in public health since it is a bacterium that is frequently linked to food-borne illnesses in humans. Some *Salmonella* serovars are responsible for outbreaks that are connected to the consumption of animal products. Cattle are connected to humans through a shared environment and the food chain as a significant source of animal protein. In Nigeria, antimicrobial medications are easily accessible for use in food-producing animals. Abattoir environments are reservoirs of foodborne bacteria like non-typhoidal *Salmonella enterica* (NTS), that have become resistant to antibiotics used for prophylaxis or treatment in animals. This study investigated the prevalence and resistance patterns of *Salmonella enterica* serovars in abattoir employees, beef cattle and abattoir environments in Abuja and Lagos, Nigeria.

**Methods:**

A total of 448 samples were collected from healthy personnel, slaughtered cattle, and abattoir environments between May and December 2020. Using Kirby-Bauer disk diffusion method, the resistance profile of NTS isolates were determined. Multidrug resistance (MDR) was considered when NTS was resistant to ≥3 antimicrobial drug classes. We performed phenotypic and genotypic characterizations of all *Salmonella* isolates including serotyping. Descriptive statistics were used to analyze the data.

**Results:**

Twenty-seven (6%) NTS isolates were obtained. Prevalence of NTS was highest in abattoir environments (15.5%; 9/58), followed by cattle (4.8%;13/272) and abattoir employees (4.2%; 5/118). A high prevalence of resistance was observed for gentamicin (85.2%; 23/27) and tetracycline (77.8%; 21/27). Whole-genome sequencing of 22 NTS showed dissemination of *aac(6’)-laa* (22/22), *qnrB19* (1/22), *fosA7* (1/22), and *tetA* (1/22) genes. Serovar diversity of NTS varied with source. *S*. Anatum, a rare serovar predominated with a prevalence of 18.2% (4/22). Chromosomal point mutations showed *ParC* T57S substitution in 22 NTS analyzed. Among 22 NTS, 131 mobile genetic elements (MGEs) were detected including insertion sequences (56.5%) and miniature inverted repeats (43.5%). Two integrating MGEs IS6 and IS21 were observed to carry the *tetA* gene + Incl-1 on the same contig in NTS originating from cattle. Rare serovars namely *S*. Abony and *S*. Stormont with MDR phenotypes recovered from cattle and abattoir environments were closely related with a pairwise distance of ≤5 SNPs.

**Conclusions:**

First report of rare serovars in Nigeria with MDR phenotypes in humans, cattle, and abattoir environments. This study demonstrates the spread of resistance in the abattoir environment possibly by MGEs and emphasizes the importance of genomic surveillance. Beef cattle may be a risk to public health because they spread a variety of rare *Salmonella* serovars. Therefore, encouraging hand hygiene among abattoir employees while processing beef cattle will further reduce NTS colonization in this population. This requires a One Health collaborative effort among various stakeholders in human health, animal health, and environmental health.

## Introduction

Antimicrobial resistance (AMR) has gained global attention due to its emergence as a public health threat in recent times. AMR has been shown to cause ten million fatalities annually; if it is not controlled by the year 2050, with 40% of these deaths occurring in the human population in Africa, which is second to Asia [1]. A high level of AMR has been detected in the human population as a result of antimicrobial overuse or abuse in food and agriculture according to the World Health Organization [2]. In Nigeria, antimicrobials are easily accessible for over-the-counter purchase without a veterinarian’s prescription for preventive and therapeutic purposes, increasing abuse and misuse by livestock farmers [3]. Thus, misuse and abuse of antibiotics promotes the emergence and spread of AMR infections in both veterinary and human medicine [4]. Acquired resistance genes (ARGs) and chromosomal point mutations have been observed in both pathogenic bacteria and the internal microbiota of exposed people and food producing animals [5]. ARGs can be transmitted on to humans and zoonotic pathogens like *Escherichia coli* and other *Salmonella* spp, as well as other Gram-negative bacteria in the gut through horizontal gene transfer [6].

As a key source of animal protein, beef cattle, one of the food-producing animals, are connected to humans through a shared environment and the food chain [7]. In Nigeria, as in many emerging economies, many families rely on beef cattle rearing as a source of income as well as a major supply of animal protein, resulting in increased beef consumption [8]. Non-typhoidal *Salmonella* (NTS) are transmitted to humans mostly through the ingestion of contaminated raw or undercooked meat or other animal products [9, 10]. NTS is the leading cause of diarrhea, infecting 550 million people annually, including 220 million in young children under five years of age [10]. Many of the instances are fatal or life-threatening [10]. Direct contact with sick animals as well as contaminated food products, especially those of animal origin, have been linked to human salmonellosis. *Salmonella* spp. spread from an animal’s intestinal tract and, contaminates meat while being processed at the abattoir [11]. The emergence of AMR bacteria such as *Salmonella* spp in humans and animals is a global public health concern that demands prompt attention [12]. NTS are routinely detected in farm and industrial settings in a wide range of food-producing animals, including poultry, swine, and cattle [13].

Pathogenic bacteria in beef cattle and the abattoir environment, such as *Salmonella* species, have developed resistance to antimicrobial drugs used for prophylaxis or treatment in food animal production [14, 15]. Antimicrobial selection pressure for bacterial drug resistance is quite significant in cattle, with a comparatively high proportion of resistant bacteria in their fecal microbiota [16]. In Nigeria, human AMR trends are comparable to those seen in animal populations and the environment [17]. Furthermore the bacteria in infected people have developed resistance to antibiotics such as penicillin, tetracycline, ampicillin, nalidixic acid, chloramphenicol, and cotrimoxazole, among others, though the relationship between these resistance patterns in humans, animals, and the environment has not been proved [18]. Although a recent study in Nigeria reported ARGs, plasmids and virulence factors in NTS isolates recovered from clinical sources, food animals and environmental sources [17].

It is therefore important to establish an AMR monitoring program in Nigeria using a One Health approach to assess the burden of AMR as well as the association between AMR patterns observed in human population, beef cattle and the abattoir environment. Our hypothesis is that slaughtered beef cattle colonized with NTS could be potential sources of resistance gene transmission along the food chain or to abattoir employees exposed due to their occupation and the abattoir environments.

Thus, this study aimed to determine the prevalence and AMR patterns of NTS in human stool, beef cattle caeca samples and abattoir environments using antimicrobial susceptibility testing and whole-genome sequencing to facilitate potential public health actions.

## Methods

### Study design and sample collection

From May to December 2020, a cross-sectional study was conducted at one abattoir in each of Nigeria’s two largest cities namely the capital Abuja located in the Northern and dry part with 3,840,000 inhabitants and Lagos in the Southern and humid part with a population of 15,946,000 [19]. For anonymity, the abattoirs were identified as A and B. A considerable number of samples were randomly collected from abattoir employees being 18 years of age, beef cattle, and abattoir wastewater using sterile containers. A total of 118 fresh stool samples were collected from selected asymptomatic healthy abattoir employees, 272 cecal contents were collected from the ceca of slaughtered beef cattle, 58 environmental samples comprising 100 ml of abattoir wastewater as well as four meat table swabs were collected at random from different points at the selected abattoirs. The samples were transported using polystyrene boxes to the Nigeria Center for Disease Control Reference Laboratory in Gaduwa, Abuja.

### Ethical considerations

The FCT Health Research Ethics Committee’s Scientific and Ethical Committee granted approval for the project (Approval Number: FHREC/2020/01/40/04-05-20). The criteria and regulations set forth by the ethics committee were followed in all the procedures. The administration of each study location was contacted for permission. Before administering the questionnaire, each eligible employee at an abattoir provided written informed consent. The gathered information was kept confidential.

### Study population and sampling

A stratified sampling technique on proportional basis was used for selection of study subjects. Employees were divided into five strata based on the nature of their job: butchers, meat sellers, livestock farmers/traders, veterinarians/para-veterinarians, and others. Sampling frames for the different strata were prepared and from each frame, abattoir employees were selected based on a table of random numbers.

The beef cattle were divided into two strata based on breed (white Fulani, Sokoto gudali, Ndama and Muturu) and gender at the time of slaughter. Cattle were randomly selected to ensure only one animal was sampled per herd based on the information provided by the owner prior to slaughter of the animal in question. Animals were selected at an interval of four until the total sample size was obtained.

Data was collected through an interviewer-administered questionnaire at the time of sample collection from animals and abattoir employees. We interviewed all abattoir employees meeting the eligibility criteria. The recruitment period for this study was 23^rd^ June– 29^th^ August 2020.

### Isolation and identification of *Salmonella* isolates

Briefly, human stool, caecum and environmental samples were pre-enriched in selenite F broth in a 1:10 sample to broth ratio at 37°C for 18–24 hours. A 10ul loop full of the enrichment broth was streaked simultaneously on Brilliant Green Agar (BGA) (Oxoid, UK) and Xylose Lysine Desoxycholate (XLD) (Oxoid, UK) media. The plates were incubated at 37°C for 24 hours [20]. Typical *Salmonella* colonies were confirmed by biochemical assays using commercially available kit Microbact GNB 24E (Oxoid, UK) according to the manufacturer’s instruction. Further confirmation was performed using the MALDI-TOF mass spectrometer (Bruker, Billerica, MA, USA). *E*. *coli* (ATCC 25922) was used as a negative control for this study.

### Antimicrobial susceptibility testing

The Kirby Bauer disk diffusion method was applied testing the antimicrobial susceptibility of *Salmonella* isolates [21] and interpreted using the Clinical and Laboratory Standards Institute (CLSI) M100 32^nd^ Edition [22]. The isolates were tested using a panel of 14 antibiotics disks (Oxoid, Hampshire, UK) of different drug classes commonly used to treat bacterial infections in humans and animals namely ampicillin (10μg), azithromycin (15μg), cefotaxime (30μg), cefoxitin (30μg), ceftazidime (30μg), chloramphenicol (30μg), ciprofloxacin (30μg), gentamicin (10μg), imipenem (10μg), meropenem (10μg), nalidixic acid (30μg), nitrofurantoin (300μg), (tetracycline (30μg), and trimethoprim-sulfamethoxazole (1.25/23.75μg). We categorized *Salmonella* isolates with intermediate breakpoints as resistant because these strains are more likely to develop resistance during the treatment course. Isolates that were resistant to three or more classes of antimicrobials were considered multi-drug resistant (MDR) [23].

### Whole genome sequencing of *Salmonella* isolates

The Noguchi Memorial Institute for Medical Research at the University of Ghana performed whole genome sequencing (WGS) of the isolates in the capacity as Regional WGS Reference Center of the UK AID Fleming Fund Regional Grant “SeqAfrica”. In brief, all *Salmonella* isolates (n = 27) from overnight culture were extracted and purified using the QIAamp DNA mini kit (Qiagen Inc. GmbH, Holden, Germany) according to the manufacturer’s recommendations. The DNA concentrations were quantified using the Qubit 4.0 Fluorometer Assay Kit (Thermo Fisher Scientific, MA, USA). The Nextera Flex Kit (Illumina Inc., San Diego, CA, USA) was used to prepare libraries according to the manufacturer’s instructions. Quantification of the libraries was performed using the 2100 Bioanalyzer System (Agilent) and Kapa Sybr Fast qPCR Kit. The pooled DNA sample was sequenced on an Illumina Miseq platform using a 2 × 300 paired-end approach (Illumina Inc., San Diego, CA, USA). Quality control was conducted on the raw sequencing reads to a Phred score of 30, a minimum read length of 50bp, and adaptors were trimmed using Trimmomatic (http://www.usadellab.org/cms/index.php?page=trimmomatic). Using the FastQC tool (https://www.bioinformatics.babraham.ac.uk/projects/fastq), we assessed the quality of reads. Thereafter, we used the resultant high-quality reads for de novo assembly using the Unicycler assembler v0.4.9 [24].

### *In silico* bioinformatics analysis

ResFinder version 4.3.3 (database version 2023-04-12/ 2023-05-03) was used to detect the acquired AMR genes and chromosomal point mutations with the identity threshold and minimum length set at 90% and 60%, respectively (http://genepi.food.dtu.dk/resfinder) [25]. The MobileElementFinder of the Center for Genomic Epidemiology (CGE) (database version 2020-06-09) was used to predict the mobile genomic elements (MGEs) linked to acquired AMR genes (ARGs) and virulence factors (https://cge.food.dtu.dk/services/MobileElementFinder/) [26]. Each AMR gene was categorized as either having no linkage or located on a MGE. The MLST profiles were assigned using the EnteroBase website https://enterobase.warwick.ac.uk/species/senterica using the seven housekeeping genes (*aroC*, *dnaN*, *hemD*, *hisD*, *purE*, *sucA*, and *thrA*). Sequence types (STs) were assigned based on allelic variations matching with 100% identity to query database. Subsequently, the *Salmonella* genomes were in *silico* serotyped using SISTR from the EnteroBase website.

### Phylogenetic analysis

Using the CSI phylogeny 1.4 tool from the CGE, the assembled genome contigs were mapped to the *Salmonella* reference genome (GenBank accession NZ_CP019413.1) to create a maximum likelihood phylogenetic tree (https://cge.food.dtu.dk/services/CSIPhylogeny/). The *Salmonella* reference genome was the best match to the collection after using the CGE K-merFinder 3.2 (database version 2022-07-11; https://cge.food.dtu.dk/services/KmerFinder/) [27]. Pairwise single-nucleotide polymorphisms (SNPs) analysis of the *Salmonella* core genome was used to assess the relatedness between the isolates and considered clonal related if two or more *Salmonella* isolates exhibited less than five distinct SNPs. The iTOL version 6 tool was applied to visualize the SNP-based phylogenetic tree (http://itol.embl.de/itol.cgi).

### Data collection and analyses

Frequencies and proportions of participants’ demographics, NTS prevalence, antimicrobial susceptibility testing results, ARGs, and MGEs were computed using R version 4.2.3 (http://www.rstudio.com/). The National Center for Biotechnology Information (NCBI) has received the raw sequencing read data for this investigation and has assigned it the project accession number PRJNA804483 (S1 File).

## Results

### Prevalence of non-typhoidal *S*. *enterica* from different sources

Out of 448 samples collected from 118 abattoir employees, 272 beef cattle and 58 abattoir environments, 27 (6%) were positive for *S*. *enterica*. For the various sample types, the prevalence of NTS in ascending order was 4.2% (5/118), 4.8% (13/272), 15.5% (9/58) in abattoir employees, slaughtered beef cattle and abattoir environments, respectively.

### Antimicrobial resistance profile of *Salmonella* isolates

No carbapenem resistance was observed among the 27 *Salmonella* isolates although a relatively high resistance level was observed for gentamicin (85.2% [23/27]) and tetracycline (77.8% [21/27]). Resistance to chloramphenicol (11.1%, [3/27]) and trimethoprim-sulfamethoxazole anti-folate combination (25.9%, [7/27]), which are the historical first-line *Salmonella* treatment, was however, infrequent, whereas resistance to ampicillin (40.7%, [11/27]) was mostly isolated from the abattoir environment (5/9), with only two human isolates exhibiting this characteristic (Table 1). Resistance to second-line antimicrobial agents for treating *Salmonella*, resistance to cephems was uncommon, with human isolates resistant to second (cefoxitin; 2/5) and third generation (cefotaxime; 1/5 and ceftazidime, 2/5) cephalosporins. In contrast, resistance to quinolones was frequent for ciprofloxacin (55.6%, [15/27]), with lower levels to nalidixic acid (22.2%, [6/27]) (Table 1).

**Table 1:** Antimicrobial resistance profiles of *Salmonella* isolates from abattoir employees, beef cattle and abattoir environments.

Drug Class	Drug	Resistance break point (mm)	Human	Cattle	Environment	Total
			n = 5 (%)	n = 13 (%)	n = 9(%)	n = 27 (%)
**Tetracyclines**	Tetracycline (TE)	≤ 11	4 (80.0)	9 (69.2)	7 (77.8)	21 (77.8)
**Folate Pathway antagonists**	Sulfamethoxazole/Trimethoprim (SXT)	≤ 10	2 (40.0)	1 (89.7)	4 (44.4)	7 (25.9)
**Penicillins**	Ampicillin (AMP)	≤ 13	2 (40.0)	4 (30.8)	5 (55.6)	11 (40.7)
**Aminoglycosides**	Gentamicin (CN)	≤ 12	5 (100.0)	11 (84.6)	7 (77.8)	23 (85.2)
**Macrolides**	Azithromycin (AZM)	≤ 12	2 (40.0)	7 (53.8)	6 (66.7)	16 (59.3)
**Phenicols**	Chloramphenicol (C)	≤ 12	1 (20.0)	1 (7.7)	1 (11.1)	3 (11.1)
**Quinolones**	Ciprofloxacin (CIP)	≤ 20	4 (80.0)	4 (30.8)	7 (77.8)	15 (55.6)
	Nalidixic acid (NA)	≤ 13	2 (40.0)	1 (7.7)	3 (33.3)	6 (22.2)
**Nitrofurans**	Nitrofurantoin (F)	≤ 14	3 (60.0)	7 (53.8)	5 (55.6)	15 (55.6)
**2**^**nd**^ **and 3**^**rd**^ **Generation Cephalosporins**	Cefoxitin (FOX)	≤ 14	2 (15.3)	0 (0)	2 (22.2)	4 (14.8)
	Ceftazidime (CAZ)	≤ 19	2 (40.0)	0 (0)	1 (11.1)	3 (11.1)
	Cefotaxime (CTX)	≤ 22	1 (7.7)	1 (20.0)	0 (0)	2 (7.4)
**Resistance to 3 or more classes of antibiotics**	MDR	n/a	4 (80.0)	10 (76.9)	8 (88.9)	22 (81.5)

Multi-drug resistance was observed in 81.5% (22/27) of *Salmonella* isolates, and 4/5 of the human isolates were MDR (Table 1). Co-resistance to the historical first-line drugs used in *Salmonella* treatment, including chloramphenicol, ampicillin, and trimethoprim-sulfamethoxazole combination, was detected in two strains: a human isolate and an abattoir environment isolate that was resistant to 7/14 antimicrobials tested.

### Whole-genome sequencing (WGS) and bioinformatic analysis

Five of the genomes did not meet the quality criteria and were omitted from further genomic analysis and characterization.

### *In silico Salmonella* serotyping and MLST

**Fig 1:**
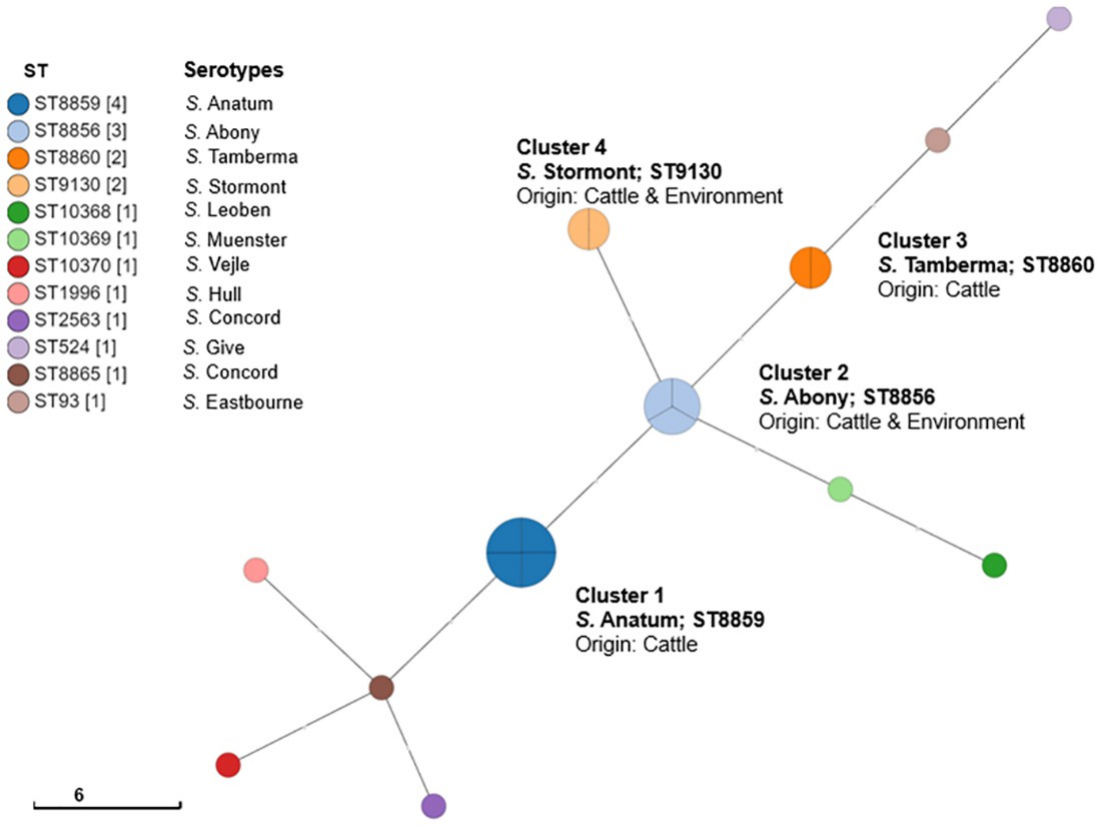
Minimum spanning tree of NTS strains used in this study. The node colors correspond to the clustering of genetically related isolates based on the multi-locus sequence types.

Three isolates of the 22 were not assigned any MLST hence they were excluded from the cluster analysis. The *in silico* serotyping and MLST of the 22 *S*. *enterica* genomes revealed a total of 12 different serovars and MLSTs from human, cattle and environmental sources including *S*. Anatum /ST8859 (n = 4), *S*. Abony /ST8856 (n = 3), *S*. Sinstorf /ND (n = 3), *S*. Concord (ST2563, ST8865) (n = 2), *S*. Stormont /ST9130 (n = 2), *S*. Tamberma /ST8860 (n = 2), *S*. Eastbourne /ST93, *S*. Give /ST524, *S*. Hull /ST1996, *S*. Leoben /ST10368, *S*. Muenster /ST10369, and *S*. Vejle /ST10370 (Table 2; Fig 1). The most prevalent serotype and MLST was *S*. Anatum/ ST8859 in isolates recovered from slaughtered beef cattle 18.2% (4/22) followed by *S*. Abony (2/22; 9.1%) and *S*. Sinstorf (2/22; 9.1%) co-predominated in abattoir environments. The serovar/ MLST diversity varied with source: the highest was observed in beef cattle at slaughter (7/12) followed by abattoir environments (5/12) with abattoir employees (3/12) having the least serovar diversity. *S*. Anatum predominated in slaughtered beef cattle with a prevalence of 18.2% (4/22). *S*. Abony (2/22; 9.1%) and *S*. Sinstorf (2/22; 9.1%) co-predominated in abattoir environments. The three abattoir employees had three different NTS serovars including *S*. Give, *S*. Vejle and *S*. Muenster. The *S*. Concord isolates originating from beef cattle at the same abattoir were both resistant to gentamicin, however, they differed by sequence type.

**Table 2:** Antimicrobial resistance profile of non-typhoidal *S*. *enterica* isolates from abattoir employees, beef cattle and abattoir environments.

Strain	Source	Location	MLST	Rare Serovars	AMR Phenotype	AMR Genotype
Sal 43	Cattle	Abuja	ST8856	*S*. Abony	AZM, TE, F	*aac(6’)-laa*
Sal 103	Cattle	Abuja	ST8860	*S*. Tamberma	AZM, TE, CN, CIP, F	*aac(6’)-laa*
Sal 294	Cattle	Lagos	ST8860	*S*. Tamberma	AZM, TE, CN, F	*aac(6’)-laa*
Sal 46	Cattle	Abuja	ST2563	*S*. Concord	CN, CIP	*aac(6’)-laa*
Sal 167	Cattle	Abuja	ST8865	*S*. Concord	TE, CN	*aac(6’)-laa*, *tet(A)*
Sal 86	Cattle	Abuja	ST9130	*S*. Stormont	AMP, C, NA, CTX, FOX, F	*aac(6’)-laa*
Sal 131	Cattle	Abuja	ST10368	*S*. Leoben	AZM, TE, CN	*aac(6’)-laa*
Sal 80	Cattle	Abuja	ND	*S*. Sinstorf	AMP, TE, CN, SXT, CIP	*aac(6’)-laa*
Sal 245	Cattle	Lagos	ST8859	*S*. Anatum	AMP, AZM, TE, CN, FOX, F	*aac(6’)-laa*
Sal 276	Cattle	Lagos	ST8859	*S*. Anatum	AMP, TE, CN, F	*aac(6’)-laa*
Sal 282	Cattle	Lagos	ST8859	*S*. Anatum	CN	*aac(6’)-laa*
Sal 286	Cattle	Lagos	ST8859	*S*. Anatum	AZM, CN	*aac(6’)-laa*
Sal 407	Environment	Lagos	ST1996	*S*. Hull	-	*aac(6’)-laa*
Sal 409	Environment	Lagos	ST93	*S*. Eastbourne	AMP, AZM, TE, CN, SXT, CIP, NA, CAZ, FOX, F	*aac(6’)-laa*
Sal 424	Environment	Lagos	ST8856	*S*. Abony	AZM, TE, CN, CIP, F	*aac(6’)-laa*
Sal 447	Environment	Abuja	ST8856	*S*. Abony	AMP, AZM, TE, CN, SXT, CIP, C	*aac(6’)-laa*
Sal 444	Environment	Abuja	ST9130	*S*. Stormont	AMP, AZM, TE, CN, SXT, CIP, NA	*aac(6’)-laa*
Sal 435	Environment	Abuja	ND	*S*. Sinstorf	TE, CN, CIP, F	*aac(6’)-laa*
Sal 448	Environment	Abuja	ND	*S*. Sinstorf	AMP, AZM, TE, CN, SXT, CIP, NA, FOX	*aac(6’)-laa*
Sal 148	Human	Abuja	ST10369	*S*. Muenster	TE, CN, CIP, NA, CAZ	*aac(6’)-laa*, *qnrB19*, *fosA7*
Sal 166	Human	Abuja	ST524	*S*. Give	CN, CIP	*aac(6’)-laa*
Sal 379	Human	Lagos	ST10370	*S*. Vejle	AMP, TE, CN, SXT, CIP, NA, CAZ, CTX, F	*aac(6’)-laa*

AMP-Ampicillin; AZM-Azithromycin; C-Chloramphenicol; CAZ-Ceftazidime; CN-Gentamicin; CTX-Cefotaxime; CIP-Ciprofloxacin; F-Nitrofurantoin; FOX-Cefoxitin; NA-Nalidixic acid; SXT-Sulfamethoxazole/Trimethoprim; TE-Tetracycline; ND-Not determined.

### Detection of resistance genes and chromosomal point mutations

Bioinformatic analysis of NTS showed dissemination of ARGs including genes that encode quinolone resistance proteins (*qnrB19* [1/22]); fosfomycin inhibitors (*fosA7* [1/22], and efflux pumps (*tetA* [1/22]).

Among the isolates that were analyzed, chromosomal point mutations were observed. The *parC* T57S substitution, a known mutation in ciprofloxacin resistance was detected in all 22 *S*. *enterica* strains analyzed, but the *gyrA* and *parE* mutations in quinolone resistance-determining regions (QRDR) were not present among the isolates. All isolates with ciprofloxacin resistance phenotype which showed mutations in *parC*, presented single amino acid substitution of threonine (T) to serine (S) and a corresponding nucleotide change from ACC to AGC. There was a concordance between ciprofloxacin resistance phenotype and genotype among the NTS isolates.

### Association of mobile genetic elements (MGEs) with AMR

**Fig 2:**
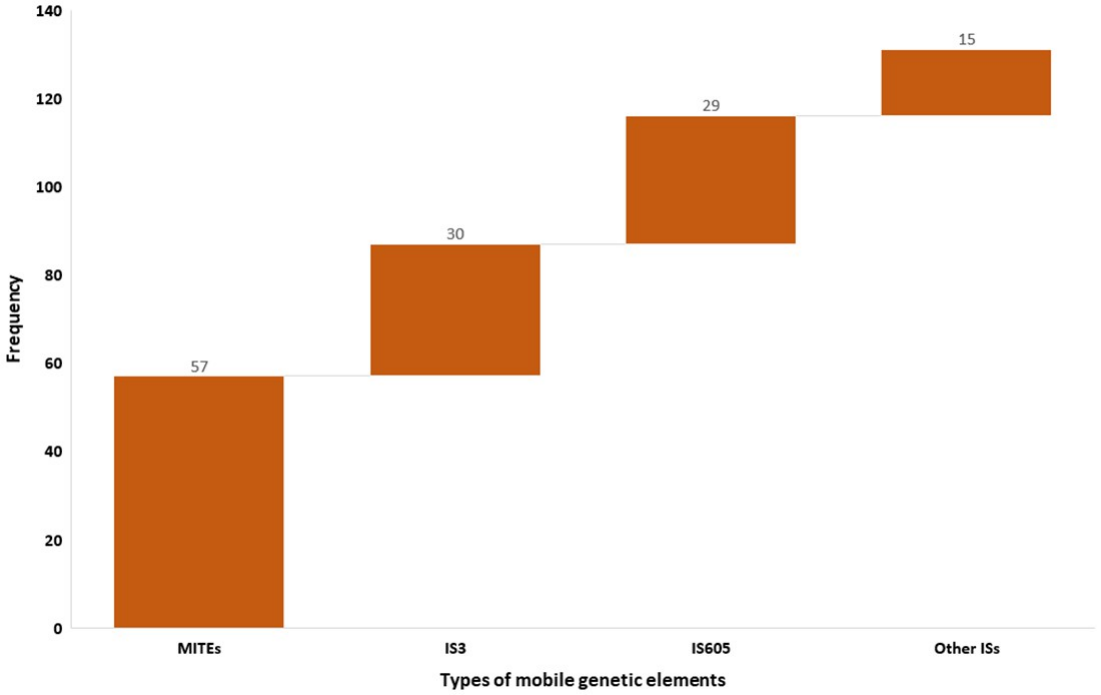
Predicted MGEs in non-typhoidal *S*. *enterica* isolates from all sources.

Isolates from all sources showed the presence of insertion sequences (ISs) and miniature inverted repeats (MITEs). As indicated in Fig 2, MobileElementFinder predicted 131 integrating MGEs in total among 22 *S*. *enterica* isolates, of which the majority were ISs (56.5%; n = 74) followed by MITEs (43.5%; n = 57). Among all predicted ISs, 40.5% (n = 30) were IS3s, followed by IS605 (39.2%; n = 29), IS630 (12.2%; n = 9), IS110 (5.4%; n = 4), IS6 and IS21 (1.4%; n = 1 each) in descending order.

Of 58 MITEs, 58.6% (n = 34), 27.6% (n = 16), and 13.8% (n = 8) were detected in *S*. *enterica* isolates from beef cattle, abattoir environments and abattoir employees respectively with 55.2% from abattoir A in Abuja and 44.8% from abattoir B in Lagos. Surprisingly no composite or unit transposons were detected. Two integrating MGEs IS6 and IS21 were observed to carry the *tetA* gene + Incl-1 on the same contig in one isolate originating from beef cattle in abattoir A. Interestingly one isolate recovered from an abattoir employee in abattoir A observed to carry a plasmid mediated quinolone resistant gene (*qnrB19*) was not located on any integrating MGE but rather on a plasmid (Col440I).

### Clustering of *Salmonella* isolates based on MLST

Clustering of the *S*. *enterica* strains based on their origin and MLST revealed four distinct clusters of 11 *S*. *enterica* strains. (Table 2; Fig 1). Of those included in the tree, eight isolates were not clustered including *S*. Concord, *S*. Leoben, *S*. Sinstorf, *S*. Give, *S*. Vejle, *S*. Muenster, *S*. Hull, and *S*. Eastbourne.

Cluster 1 with *S*. Anatum (ST8859) comprised of four *Salmonella* isolates all originating from slaughtered beef cattle in Abattoir B (Lagos). Cluster 2 with *S*. Abony (ST8856) consisted of three *Salmonella* isolates originating from cattle (one strain from Abattoir A) and abattoir wastewater (two strains, one each from Abattoir A and B). Cluster 3 with *S*. Tamberma (ST8860) included two *Salmonella* isolates originating from cattle while Cluster 4 with S. Stormont (ST9130) consisted of two strains (one from cattle and the other from abattoir wastewater in Abattoir A).

### Phylogenetic Single Nucleotide Polymorphism (SNP) based analysis

**Fig 3:**
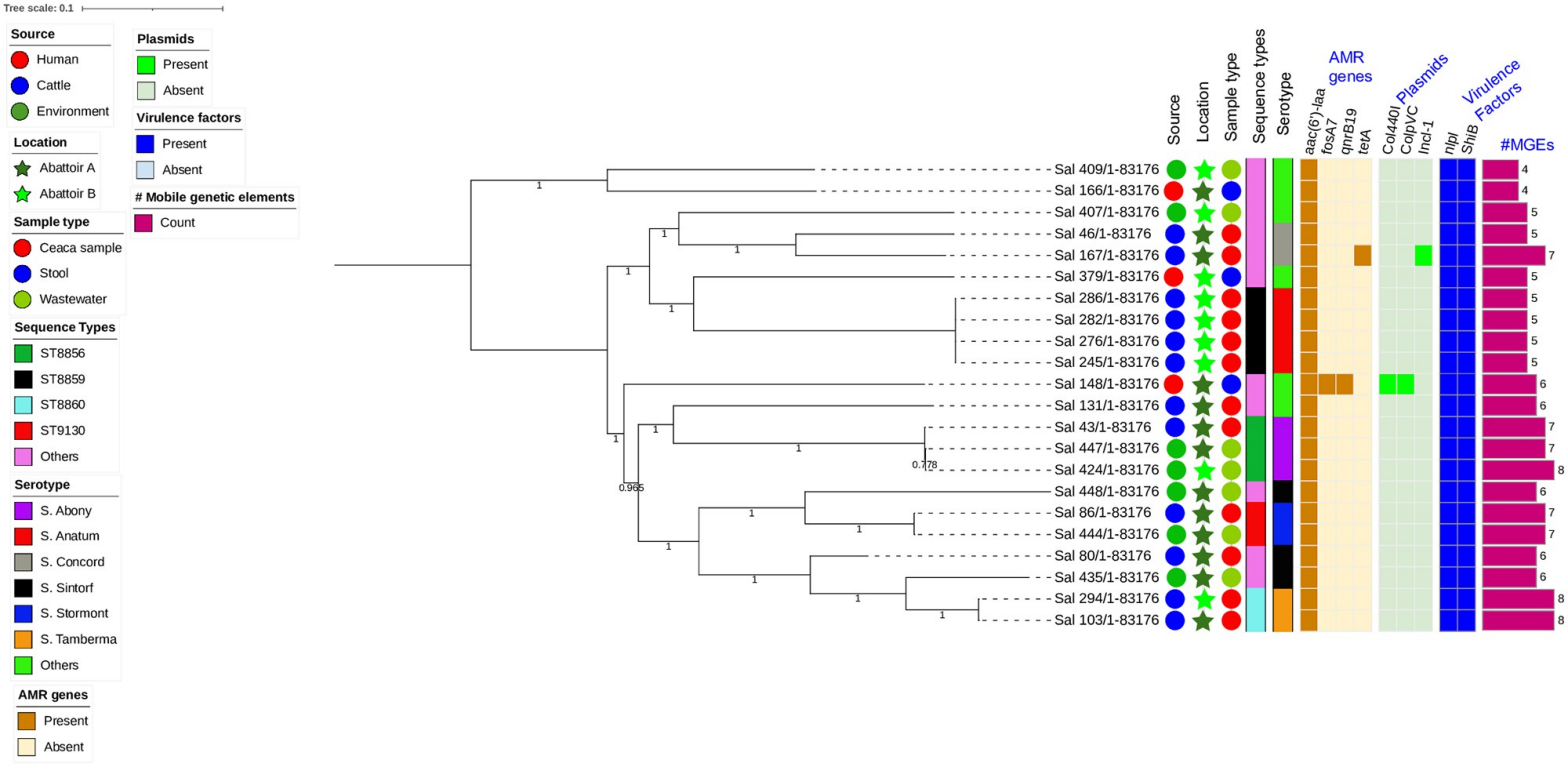
SNP-based phylogeny of non-typhoidal *S*. *enterica* isolates from abattoir employees, beef cattle, and abattoir environments in Abuja and Lagos, Nigeria. SNP-based maximum likelihood phylogeny of *S*. *enterica* isolates visualized in iterative Tree of life tool (iTol). The tree was rooted in a reference isolate *S*. *enterica* strain with NCBI Gen bank accession.

Many of the isolates generally formed individual lineages, however some isolates from the same source showed genetic relatedness with pairwise SNP differences of ≤5 (Table 3). For example, two pairs of NTS isolates with MDR phenotypes from beef cattle and abattoir wastewater were closely related with a pairwise distance of ≤5 SNPs. The phylogenetic SNP analysis (comprising maximum-likelihood phylogenetic tree; pairwise SNP matrix) revealed no clonal cluster shared by human and beef cattle *S*. *enterica* isolates (Fig 3). All the human isolates with different serovars were genetically diverse showing individual lineages with pairwise distance of over 27646 SNPs (S1 File). Plasmid replicons were only detected in one *S*. Concord isolate recovered from beef cattle and one MDR *S*. Muenster isolate recovered from an abattoir employee. These plasmids were observed to be harboring ARGs correlating with the observed AMR phenotypes: Incl-1 + *tetA* (tetracycline) and Col440I + *qnrB19* (ciprofloxacin and nalidixic acid).

**Table 3:** Clonal relationships between non-typhoidal *S*. *enterica* isolates from different sources.

Clonal relationship	Sample IDs	Rare Serovars	Source	SNP difference
A	Sal 245 & Sal 276	*S*. Anatum	Cattle	0
B	Sal 245 & Sal 282	*S*. Anatum	Cattle	0
C	Sal 245 & Sal 286	*S*. Anatum	Cattle	0
D	Sal 103 & Sal 294	*S*. Tamberma	Cattle	1
E	Sal 43 & Sal 447	*S*. Abony	Cattle & Environment	1
F	Sal 424 & Sal 447	*S*. Abony	Environment	2
G	Sal 43 & Sal 424	*S*. Abony	Cattle & Environment	3
H	Sal 86 & Sal 444	*S*. Stormont	Cattle & Environment	5

Clonal relationships A, B, C, D and F occurred in NTS isolates originating from the same source while E, G and H occurred in isolates from different sources. These isolates were closely related having a pairwise SNP difference of < 5

## Discussion

Many investigations around the world have found that non-typhoidal *S*. *enterica* (NTS) isolated from food animals, especially beef cattle, are frequently resistant to antimicrobials [12, 14, 17, 28]. Cattle have been implicated as a likely source in the transmission of NTS to humans via the food chain [9, 10, 13]. The hazards of people working in proximity with these food animals and the possibility of being colonized with NTS have however, not been adequately explained.

The present study investigated the prevalence of NTS in abattoir environments, slaughtered beef cattle, and abattoir employee populations. Our research demonstrated that NTS are prevalent in the abattoir environment where beef cattle are slaughtered for food, acting as a reservoir of resistant bacteria, and posing a health risk to these abattoir employees.

In this study, the prevalence of NTS was much higher than that of a recent study conducted in one of our study locations (Lagos, Nigeria) that reported a prevalence of 2.2%, 0.9% and 12.0% in isolates recovered from cattle, human stool and environmental samples, respectively [17]. Another study among poultry workers in a different location in Nigeria reported a much higher prevalence of 23.4% when compared to our study results among abattoir employees, a slightly different population [29]. The higher NTS prevalence observed in the present study may have been a result of the poor sanitary conditions of the abattoirs, especially because NTS has been reported to contaminate meat during processing at abattoirs [11]. Although a higher prevalence of 6.7% (3/45) was reported in beef in a similar study in Ghana [30] while a much lower overall prevalence of 2.8% (23/830) was reported in cattle carcass, abattoir workers and abattoir environment in Cameroon [31]. The differences in prevalence observed when compared to our study may have been because of the varying sample sizes in these studies. It is important to note that other *Salmonella* spp have been isolated from food animals in Africa including chickens, ducks, cattle, pigs, goats and sheep in addition to humans and the environment [32, 33].

All 27 NTS isolates in the present study were susceptible to imipenem and meropenem at phenotypic characterization, and this consistent with findings of a similar study carried out in Lagos, Nigeria [17]. The results of this study showed that the NTS isolates all together had high phenotypic resistance to aminoglycosides, tetracycline, macrolides, quinolones, nitrofurans and penicillin. Aminoglycosides, however, accounted for most of the resistance determinants detected among NTS isolates in the present study, and this is consistent with the reports of others [17, 34]. A previous study conducted in Nigeria, isolated *Salmonella* spp. from febrile patients in Lagos hospitals that were resistant to third generation cephalosporins with resultant *bla*_*CTX-M*_ and *bla*_*CTX-M-3*_ genes detected by polymerase chain reaction (PCR) [35]. Although our study did not detect any of these genes, WGS has the potential to cover so many gene targets at the same time allowing for the simultaneous detection of all genetic elements in the genome, including known and novel AMR genes when compared to PCR which often targets specific resistance mechanisms [36].

Phenotypic resistance to quinolones was frequent especially for ciprofloxacin, but uncommon for nalidixic acid. A high level of resistance of NTS isolates to ciprofloxacin is consistent with the literature [17, 29, 34, 37, 38]. This is not surprising, as quinolones, which are medically important antimicrobials in human health, have been reported to be used as growth promoters in the livestock industry in Nigeria and may have contributed to the emergence of NTS resistant to ciprofloxacin [39]. Chromosomal mutations in the QRDR of *parC* (T57S) reported to cause clinical resistance in *Salmonella* species was observed in all 22 NTS isolates and this is in agreement with reports of other related studies conducted in Nigeria [17, 37, 38] and other locations [9, 34]. This is a known mutation conferring resistance in *Salmonella* spp isolates exhibiting low ciprofloxacin minimum inhibition concentrations as reported by other studies in recent times [40, 41].

The MDR phenotype was observed in majority of the NTS isolates, however only one isolate *S*. Muenster recovered from an abattoir employee had the MDR genotype with resistance determinants to quinolones (*qnrB19*) and fosfomycin (*fosA7*). A similar MDR pattern has been reported among *S*. Brandenburg isolates in Brazil [42].

Whole genome sequencing revealed eight rare serovars of NTS isolated from human, cattle and environmental sources including *S*. Abony; *S*. Sinstorf; *S*. Stormont; *S*. Tamberma; *S*. Leoben; *S*. Hull, *S*. Vejle, and *S*. Eastbourne. These serovars are rare based on the reports of previous surveys [43]. In addition, four common serovars, namely *S*. Anatum; *S*. Concord; *S*. Give; and *S*. Muenster were also detected. To our knowledge, except for *S*. Abony, *S*. Give, *S*. Muenster, and *S*. Eastbourne, the remaining serovars detected in this study had never been previously characterized in Nigeria, either from humans, beef cattle or the environment. According to the origin of the NTS isolates, the serovar diversity varied; the highest was found in slaughtered beef cattle, followed by abattoir environments, with abattoir employees having the lowest serovar diversity.

Interestingly, serovar *S*. Anatum was the dominant serovar in this study originating from beef cattle (18.2%). Our findings are consistent with a similar study conducted in Australia that reported a higher prevalence (27.9%) of *S*. Anatum in beef cattle ready for slaughter [44]. This serovar has also been reported to be prevalent in cattle faeces and lymph nodes [45, 46]; slaughtered pigs [47]; ground beef as well as in humans [48]. The *S*. Abony with MDR phenotype which was isolated from cattle and abattoir wastewater has been reported by a similar study from related sources in Nigeria further supporting our claims [17]. Although the MDR *S*. Abony detected in our study did not harbor any plasmid-mediated AmpC β-lactamase gene, a similar study conducted in Brazil isolated AmpC resistant *S*. Abony from food sources [43]. The *S*. Sinstorf was detected in NTS isolates from cattle, and the abattoir environment with the MDR phenotype in the present study. It is interesting to note that *S*. Sinstorf carrying mcr-1 gene was reported in diarrhea patients in China but also isolated from imported ducks and chickens in Egypt thus indicating the diversity of this NTS serotype [49, 50].

*S*. Muenster which was detected in *Salmonella* isolates from an abattoir employee in the present study has been previously isolated from pigs and chickens in Nigeria [37, 38, 51]; humans in Senegal [52]; imported ducks in Egypt [50] and cattle ready for slaughter at abattoirs in Australia [44]. Furthermore, serovar *S*. Give was detected in NTS isolates from an abattoir employee in Abuja although similar studies conducted in Nigeria reported this serotype in isolates recovered from ill humans, cattle and the environment [17, 53]. This serotype has also been detected in NTS isolates from chickens in Nigeria [51]. This is the first report of serovar *S*. Vejle in NTS isolates from an abattoir employee in Nigeria. A recent study conducted in Egypt detected this serovar in NTS isolates recovered from imported chickens [54].

Our results show that the *S*. Concord serovar was detected in isolates resistant to gentamicin and originating from slaughtered beef cattle but of different sequence type. Previously, *S*. Concord has been isolated from humans in Ethiopia and implicated in foodborne outbreaks. Although *S*. Concord in this study was not MDR, others have reported that in Ethiopia, this serotype is polyphyletic and diverse in nature spanning several lineages which are mainly MDR [55]. Other serovars with MDR phenotypes including *S*. Leoben and *S*. Eastbourne detected in NTS isolates in the current study have also been documented in slaughtered cattle and pigs [46, 47, 53]. Thus, these serovars may be emerging NTS serovars in Nigeria although not much is known about their potential to cause disease in humans. Although the present study detected *S*. Tamberma in NTS isolates originating from cattle, this serovar has been reported in clinical isolates resistant to tetracycline in Burkina Faso [56].

ARGs were categorized as being related to integrating MGEs if they were carried by the MGEs thus having the potential to be mobilized. Interestingly, two integrating MGEs IS6 and IS21 were observed to carry the *tetA* gene + Incl-1 on the same contig in one isolate originating from beef cattle in abattoir A. Tetracycline resistance genes were also carried on MGEs in *Salmonella* isolates originating from pigs further supporting our claims [26]. Most of the ARGs carried by integrating MGEs were mostly carried by insertion sequences and probably due to its propensity to organize into IS arrays which have been crucial for spreading ARGs among Gram-negative bacteria as reported by others [26, 57]. Surprisingly, our results did not detect the presence of composite or unit transposons in the NTS isolates. Other studies have reported that NTS strains can mediate the transfer of resistance gene through a number of transpositional mechanisms [26, 58]. Although these studies have reported fewer numbers of transposons when compared to insertion sequences in NTS isolates from other sources [26, 55].

The Col440I plasmid carrying *qnrB19* was detected in one NTS isolate originating from an abattoir employee. Studies have shown that the Col440I plasmid plays an important role in the dissemination of *qnrB19* especially in NTS and other members of Enterobacteriaceae [17, 37, 39]. Only one of our NTS isolates originating from beef cattle harbored the IncI-1 plasmid carrying *tetA*, despite the fact that they are widely distributed in *Salmonella* species [59], which is consistent with the results of a similar study in Cameroon [31].

Whole genome phylogenetic analysis demonstrates most of the NTS isolates were genetically diverse. The isolates clustered together in phylogenetic analysis based on their source and serotypes. Genetical diversity showing individual lineages with pairwise distance of over 27646 SNPs was observed in all the human isolates. Most of the clonal relationships observed in the current study were in NTS isolates originating from identical sources., some NTS isolates with MDR phenotypes from beef cattle and abattoir environments were however, closely related with a pairwise distance of less than 10 SNPs. Although this does not translate to direct transmission from beef cattle to abattoir environment, it is likely possible. Many studies have also documented the genetic diversity of NTS serovars as well as the possibility of clonality shared between isolates from different sources, hence supporting our claims [17, 37, 46, 51].

It is important to note that the study is limited by a small number of NTS isolates recovered from all the different sources probably because one caecal sample was evaluated per beef cattle and one stool sample per abattoir employee instead of multiple samples which enhances isolation of *Salmonella* species. It was also difficult to establish the risk involved for abattoir employees because of the small number of NTS isolates recovered from the samples. Furthermore, we did not have access to information on animal husbandry and antibiotic usage in the slaughtered cattle for the present study.

## Conclusion

In this study, MDR NTS isolates were observed to be prevalent amongst abattoir employees, beef cattle, and abattoir environments. The highest resistance rates among MDR NTS isolates were observed to aminoglycosides, tetracycline, macrolides, quinolones, nitrofurans and penicillin which are classes of antimicrobials commonly used in veterinary practice in Nigeria. In this investigation, many rare *Salmonella* serovars were isolated from slaughtered cattle, emphasizing the importance of genomic surveillance, and highlighting the need for multi-sectoral collaboration to stop the transmission of bacterial illnesses from food animal products to humans. Beef cattle may be a risk to public health because they spread a variety of *Salmonella* serovars, many of which are uncommon and may be a source of human salmonellosis in the region. Therefore, encouraging hand hygiene among abattoir employees while processing beef cattle will further reduce NTS colonization in this population. It is also recommended that the responsible government agencies take targeted control actions against newly emerging serovars and continuously monitor and control how antimicrobials are used in food animal production. This requires a One Health collaborative effort among various stakeholders in human health, animal health, environmental health, and policy makers.

## Supporting information

S1 File This file contains the metadata for the *Salmonella enterica* isolates from humans, beef cattle and abattoir environment. The metadata comprises the NCBI sequence SRR, AMR phenotype, resistance genes, mobile genetic elements, and SNP distance matrix.
(XLSX)
